# Investigating the probability of establishment of Zika virus and detection through mosquito surveillance under different temperature conditions

**DOI:** 10.1371/journal.pone.0214306

**Published:** 2019-03-28

**Authors:** A. Ryan Tramonte, Rebecca C. Christofferson

**Affiliations:** 1 Department of Pathobiological Sciences, School of Veterinary Medicine, Louisiana State University, Baton Rouge, Louisiana, United States of America; 2 Center for Computation and Technology, Louisiana State University, Baton Rouge, Louisiana, United States of America; Faculty of Science, Ain Shams University (ASU), EGYPT

## Abstract

Because of the increasing threat that Zika virus (ZIKV) poses to more sub-tropical area due to increased global travel, there is a need for better understanding of the effect(s) of temperature on the establishment potential of ZIKV within these subtropical, temperate, and/or seasonal *Ae*. *aegypti* populations. The first step to determining risk establishment of ZIKV in these regions is to assess ZIKV's ability to infect mosquitoes at less tropical temperatures, and thus be detected through common surveillance programs. To that end, the effect of two rearing temperatures (RT) and extrinsic incubation temperatures (EIT) on infection and dissemination rates was evaluated, as well as the interactions of such. Total, there were four combinations (RT24-EIT24, RT24-EIT28, RT28-EIT24, RT28-EIT28). Further, a stochastic SEIR framework was adapted to determine whether observed data could lead to differential success of establishment of ZIKV in naive mosquito populations. There was no consistent pattern in significant differences found across treatments for either infection or dissemination rates (p>0.05), where only a significant difference was found in infection rates between RT24-EIT24 (44%) and RT28-EIT24 (82.6%). Across all temperature conditions, the model predicted between a 76.4% and 95.4% chance of successful establishment of ZIKV in naive mosquito populations under model assumptions. We further show that excluding the maximum observed infection and dissemination rates likely overestimates the probability of local establishment of ZIKV. These results indicate that 1) there is no straightforward relationship between RT, EIT, and infection/dissemination rates, 2) in more temperate climates, ZIKV may still have the ability to establish in populations of *Aedes aegypti*, 3) despite an overall lack of significant differences in infection/dissemination rates, temperature may still alter the kinetics of ZIKV within the mosquito enough to affect the likelihood of infection establishment and detection within the context of mosquito surveillance programs, and 4) both the temporal and magnitude qualities of vector competence are necessary for parameterization of within-mosquito virus kinetics.

## Introduction

Zika virus (ZIKV) emerged as a public health emergency in 2016 after a steady migration eastward from Africa through the South Pacific [1, 2]. As ZIKV spread quickly across the tropics of the Western Hemisphere, the primary mosquito vector was demonstrated to likely be *Ae*. *aegypti* which has efficiently transmitted dengue virus (DENV) in the region for decades [3, 4]. The ecology of ZIKV is similar to that of DENV and chikungunya (CHIKV) owing to the shared primary vector and transmission of these three viruses has overlapped in several instances [5–7]. A major reason these viruses have not emerged with the same intensity in more sub-tropical and temperate areas is because the distribution of *Ae*. *aegypti* is restricted to warmer, humid climates [8]. However, there have been instances of seasonal transmission of DENV, CHIKV, and ZIKV in sub-tropical and even temperate areas [9–11]. Additionally, the CDC recently updated its predicted range for the principle ZIKV vectors, which shows a significant portion of the Eastern United States is at risk for at least a “likely” presence of *Ae*. *aegypti* [12].

This estimated distribution shows that the mosquito is likely to have at least some presence in subtropical regions, where there is a probability that development and potential viral incubation temperatures would be in the ranges of 24°C and 28°C. Indeed, a recent study found that *Ae*. *aegypti* were able to become infected at moderate rates at both 18°C and 27°C by 14 days post infection (dpi) suggesting that the establishment of ZIKV in mosquito populations at lower temperature is possible [13].

Following the emergence of ZIKV in the Western Hemisphere, there was a significant number of returning travelers to the United States that were positive for the virus [8, 14–18]. Detection of local transmission in Miami, Florida and Cameron County in South Texas was always based on presentation and diagnosis of a patient with no travel history [11, 19], and mosquito control programs responded to human cases (travel or local) with increased, targeted surveillance of populations. This and other data-driven means of refining mosquito surveillance practices is not uncommon, as implementation of large-scale arbovirus-specific testing procedures is not a trivial process [20–23]. The temperature limitations on mosquito and virus transmission capabilities could be used to inform seasonality of any ZIKV mosquito pool testing, should it become a standard part of local programs. But the interplay ZIKV kinetics within the mosquito, temperature, and surveillance detection has not been rigorously addressed.

Additionally, it has been demonstrated that rearing temperatures can affect the infection and dissemination of arboviruses through *Ae*. *aegypti* [24–26]. However, the results of these studies combined do not immediately suggest a clear uni-directional relationship between temperature at different life stages and vector competence and indicate that these effects still need characterization [27, 28]. On the other hand, higher temperatures during the extrinsic incubation period (EIP) are almost uniformly associated with higher rates of infection and dissemination in many arboviral systems [13, 29, 30].

This study focused on the interplay of rearing temperature (RT) and extrinsic incubation temperature (EIT) at temperatures at the lower limit of permissive seasonality (24°C and 28°C) to determine whether there was an effect on the ability of ZIKV to establish in mosquito populations and subsequently be detected by mosquito surveillance efforts. We define “establishment” as the process of introduction of ZIKV by infectious humans introduced into a naive population of mosquitoes, the contact between an introduced infectious human and a naive mosquito, the successful up-take of virus by at least one mosquito, and subsequent successful midgut infection of that (at least) one mosquito. Thus, a mathematical model was employed to determine the differential probabilities of at least one mosquito in a naïve population becoming infected and subsequently being detected using a pool-positive method based on temperature treatments. In addition, we model the probability that a mosquito will have developed a disseminated infection–a precursor to transmissibility–but explicitly specified the model not to allow for transmission as we are interested in *only* the first generation of mosquito infection. That is, the initial infection of the naive mosquito population by a finite group of introduced, infectious humans without any continuation of the transmission cycle. This constitutes a critical first step in establishment of potential autochthonous transmission chains. Further, whole-body mosquito pool testing is the most common method of detecting arboviruses in resident mosquito populations under usual surveillance programs in the United States. Therefore, the first indication of local ZIKV activity is based on detecting a mosquito that has developed a midgut infection.

## Materials and methods

### Rationale

The temperatures of 24°C and 28°C were chosen based on 1) recent literature that defined the lower limits of optimal temperature at 24°C [31], 2) evidence that human ZIKV cases did occur during periods where temperatures were between 28°C and 24°C [32], and 3) our intention to put these results into the context of defining the lower limits of temperature that relate to seasonal mosquito surveillance.

### Mosquitoes and virus

*Aedes aegypti* mosquitoes (Rockefeller strain) were originally obtained from Dr. Daniel Swale of the LSU Entomology department. The PRVABC59 strain of ZIKV was used and was originally obtained from the CDC originally isolated from a patient in Puerto Rico [33]. Viral titers of fresh (not frozen) ZIKV (passage 4 in Vero cells) were confirmed by qRT-PCR and matched (~8 x 10^7^ PFU/mL) across same-day oral feedings using whole bovine blood with Alsevers (Hemostat Laboratories, Dixon, CA) and the Hemotek membrane feeding apparatus (Discovery Workshops, UK) as in [2, 34].

### Rearing and oral exposure of mosquitoes

Mosquitoes were allowed to hatch at one of two temperatures (24°C or 28°C) and kept at this temperature until they were exposed to an infectious blood meal at 3–5 days post emergence. This is hereafter referred to as the rearing temperature (RT). After exposure to ZIKV, fully engorged females were sorted into clean cartons. Half of these were left at their original RT for the extrinsic incubation period while the other half were moved to the opposite temperature. This temperature is the extrinsic incubation temperature (EIT). Thus, there were a total of 4 treatments (total sample size for each treatment is given in parentheses and per DPI in S1 Table): RT28-EIT28 (n = 51), RT28-EIT24 (n = 77), RT24-EIT28 (n = 52), and RT24-EIT24 (n = 72). A detailed description of the experimental design and process is depicted in S1 Fig.

### Sampling, processing, and detection of ZIKV from mosquitoes

To quantify the rates of infection over time, mosquitoes were sampled on days 7, 10, and 13 post-exposure and tested for the presence of virus in the abdomens. Legs were also tested to determine dissemination rates, which is the precursor for transmission but does not necessarily indicate transmissibility. These time points were chosen based on studies that have demonstrated the timing of ZIKV infection within *Ae*. *aegypti* mosquitoes [35, 36]. Briefly, mosquitoes were cold anesthetized and the legs separated from the bodies and placed into 2mL tubes with 900 μl of BA1 and two steel-coated BBs. Samples were then homogenized using a TissueLyzer (Qiagen), centrifuged at 4,000 rpm for 4 minutes, frozen at -80°C. Samples were thawed and RNA extracted using the KingFisher (ThermoFisher) robot and the Ambion MagMax viral isolation kit (ThermoFisher), as per manufactuer’s instructions.Samples were tested for the presence of ZIKV RNA via qRT-PCR using the SuperScript III One-Step RT-PCR System with Platinum Taq DNA Polymerase (Life Technologies) on the Roche LightCycler 480 system using primers and probes previously published [37, 38]. Samples with a CT of <35 were considered positive, while samples with a CT of 35 or greater were inoculated onto Vero cells and the supernatant tested 4 days later. At this point, CT<35 was indicative of a positive sample, but a second CT value of 35 or higher was indicative of a negative sample.

### Statistical analysis of experimental data

Data were analyzed using RStudio (version 1.1.383, with base R version 3.4.3). Mosquito infection and dissemination rates were compared daily by chi-square test for equivalency of frequencies using the R command prop.test (with continuity correction, [39]). Infection rates were calculated as the number with a positive body divided by total number exposed. Dissemination rates were first calculated as the number with positive legs divided by the total number tested for a measure of population-level dissemination, and then as the proportion of disseminated infections out of total positive bodies (infected). Presence of virus in the legs indicates that the virus has gotten out of the midgut, the first within-mosquito barrier to transmission.

### Modeling the temporal processes of infection and dissemination

To further describe these processes, the data was fit using a non-linear least squares model, to determine the best exponential fit (R version 3.4.3), as these processes have been described as following an exponential distribution [40]. An origin at (0,0) was assumed. Briefly, the exponential relationship was described as:

$$p_{inf}=1-exp(-\lambda_{inf}*dpe)$$

$$p_{diss}=1-exp(-\lambda_{diss}*dpe)$$

where p_inf_ is the proportion of samples with positive bodies, λ_inf_ is the rate parameter of the exponential cumulative distribution function (CDF); p_diss_ is the proportion of mosquitoes that developed a disseminated infection given they were infected (disseminated/infected); λ_diss_ is the rate parameter fit to the disseminated/infected data; and dpe is the day post exposure corresponding to each value p [40]. The values of p_inf_ and p_diss_ were empirically obtained from the experiments described above and binomial 95% confidence intervals calculated. The values of λ_inf_ and λ_diss_ were estimated were estimated using the nls function in R and bootstrapped confidence intervals were obtained using the confint function from the ‘nlstools’ package.

The resulting fits were assessed via Kolmogorov–Smirnov test for goodness of fit by comparing a randomly generated exponential CDF with the parameter estimates from the experimental data (λ_inf_ and λ_diss_) to the data itself and all estimated fits were deemed “good” as p>0.05, indicating no rejection of the null hypothesis that no differences exist between the two distributions. Modeling these parameters with CDFs assume an asymptote of 1, we then weighted the distribution with the maximum detected positivity (p_inf.max_ and p_diss.max_) from each group which sets the asymptote at p_max_ for each process. For comparison, we include the model results when p_inf.max_ and p_diss.max_ are excluded and the infection and dissemination rates are allowed to asymptotically approach 1.

Subsequently, a stochastic, SEIR compartmental model was modified from [41]. Briefly, the simulations implemented the tau-leap approximate to Gillespie’s algorithm with a 0.125 days time step [41, 42]. The model incorporated the values of λ_inf_ to describe the average rate of movement of mosquitoes from the exposed to the infected class, and λ_diss_ describes the rate of movement from infected to the disseminated class [40]. These rates of movement were further weighted by the maximum proportion of infected or disseminated mosquitoes so that exposed→infected was defined as p_inf.max_*λ_inf_ and infected → disseminated was defined as p_diss.max_*λ_diss_. Transition states and movement between are given in S2 Table.

**Fig 1:**
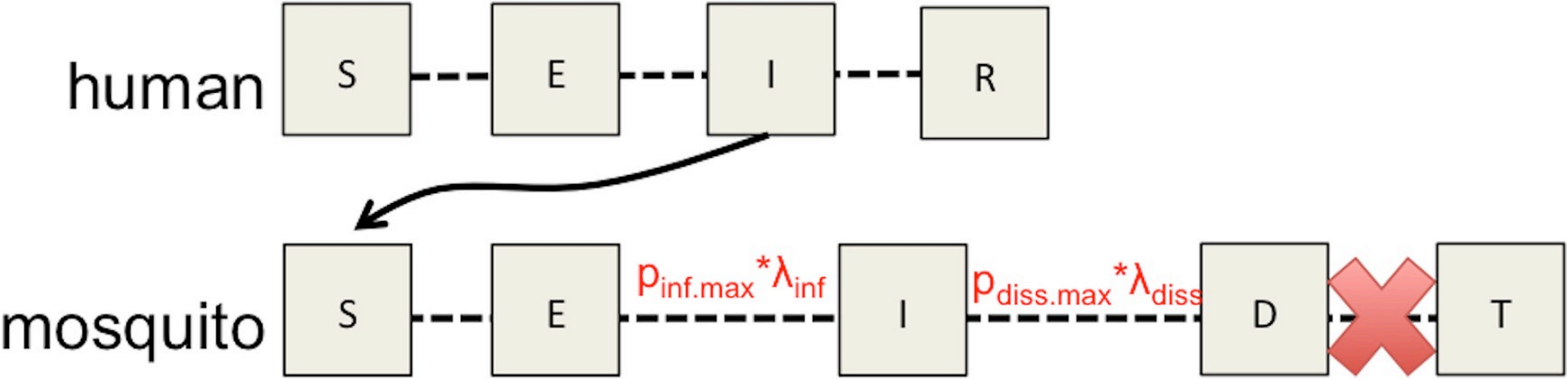
Model schematic demonstrating how experimental data informs the parameterization of a model to simulate the probability of ZIKV establishing infection in at least one mosquito and predicting the probability of at least one disseminated infection following introduction into a naïve population of *Ae*. *aegypti*.

Because the purpose of this exercise was to describe the differential potential of ZIKV to establish in mosquitoes at different temperature profiles, and the probability of detection in mosquito pools (i.e. at least one infected mosquito), the model did not allow mosquitoes to transmit, and the model was run for a total of 30 days (Fig 1). That is, the final output is number of mosquitoes exposed, infected, and disseminated following a primary, single introduction of five infectious humans (e.g., returning travelers) and accounts for a single generation of infection (human → mosquito). Subsequently, the probability of ZIKV establishing infection in or disseminating through at least one mosquito was calculated as the number of simulations with at least one infected or disseminated mosquito (respectively) divided by the total number of simulations. Model details, including other parameter values and descriptions, are given in the Supplemental Information, and their relation to transition states in S2 Table. A total of 500 simulations per treatment were run. The model was run again without the inclusion of the weight parameters (p_inf.max_ and p_diss.max_) to determine whether inclusion of this parameter would significantly affect the outcomes of the simulations.

### Determination of mosquito pool sensitivity

As part of the motivation of this study was to determine the detection of ZIKV by mosquito surveillance using mosquito pool positivity, we conducted a study to determine the sensitivity of mosquito pools of size 100 mosquitoes to detect a single positive ZIKV mosquito. Briefly, mosquitoes were exposed as above and incubated at 28°C for 6–7 days post exposure. (NOTE: These mosquitoes were not included in any of the analyses above.) Mosquitoes were freeze killed and the legs removed. Bodies were kept intact in tubes and stored at -80°C. Legs were tested for positivity to ZIKV as above. If a leg sample was found to be positive, then the intact abdomen was placed into a 2 mL tube with 99 unexposed females for a pool size of 100 total to determine whether a single mosquito in a pool of this size would be detected through surveillance. This is consistent or above the normal size of pools for mosquito surveillance testing [43] (personal communication, Tarra Harden, Louisiana Animal Disease Diagnostic Laboratory, Mosquito Pool Testing). Mosquito pools were processed as above and tested for the ZIKV. There were a total of 8 pools of 100 total mosquitoes (99 uninfected, 1 infected per each pool) and each pool was tested in duplicate. A single positive was coded as “positive”. An additional pool with 100 uninfected mosquitoes was used as a pool negative control as an additional negative control.

## Results

### Effect of RT-EIT on infection and dissemination rates

**Fig 2:**
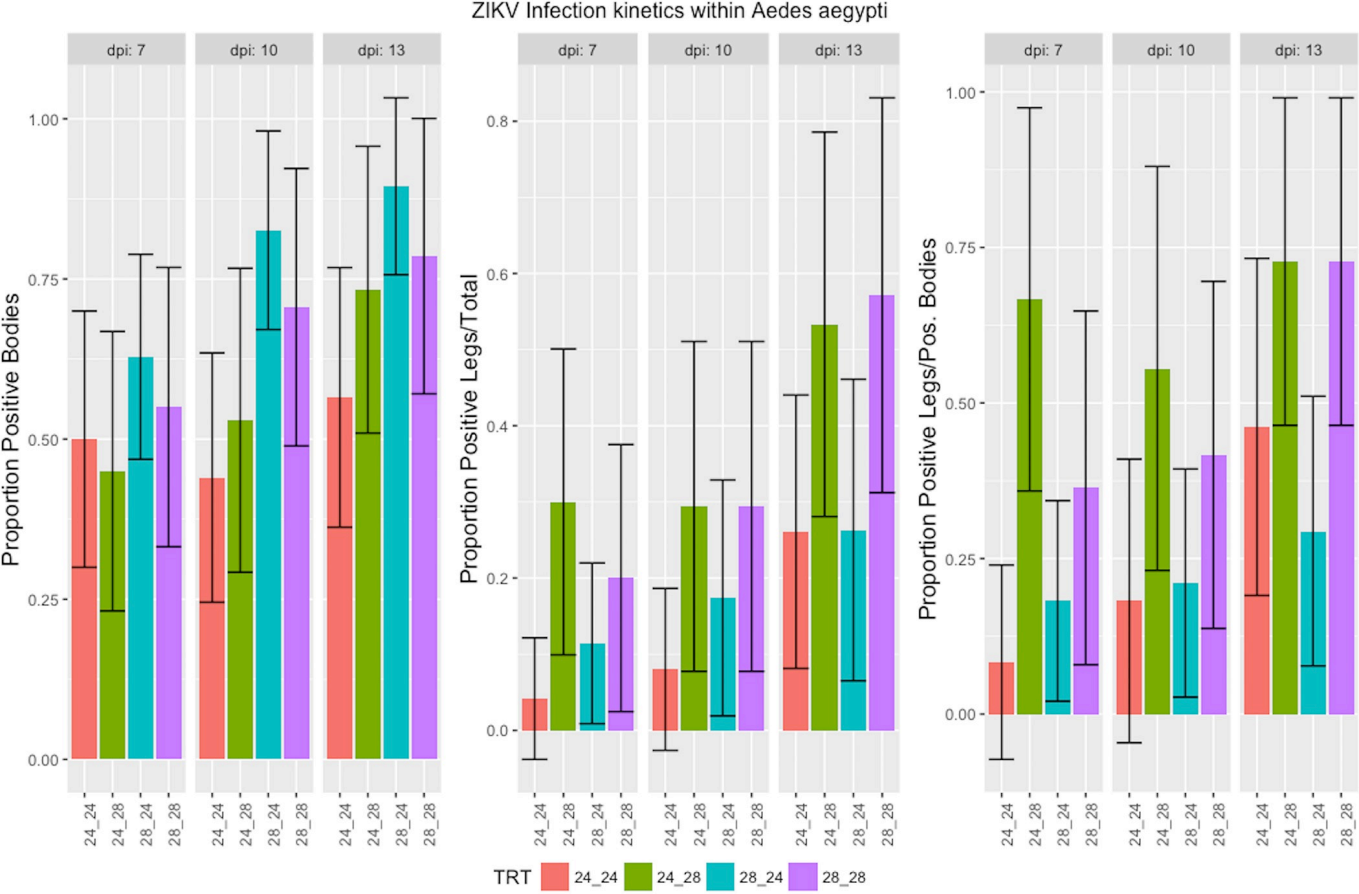
Proportion of mosquitoes that became (Left) infected with Zika, and those that developed a disseminated infection calculated as (Middle) disseminated/total and (Right) disseminated/infected. Statistical significance was determined via Chi-square test for equal proportions (α = 0.05). Error bars represent the binomial 95% confidence intervals of the proportions.

The infection and dissemination rates are shown in Fig 2 and in S1 Table. At days 7- and 13-post infection, there was no significant difference in either the ability of ZIKV to infect or to disseminate through mosquitoes among temperature combinations (p>.05). At day 10, there was a significant difference in the proportion of infected mosquitoes, and pairwise comparisons identified this difference between RT24-EIT24 (44%) and RT28-EIT24 (82.6%). However, there was observed no significant difference in the ability of ZIKV to escape the midgut among the temperature combinations. When the proportion of disseminated mosquitoes was calculated as the proportion positive divided by infected mosquitoes, there was a significant difference at day 7 dpe between RT24-EIT24 and RT24-EIT28 (p = .019) and between RT24-EIT28 and RT28-EIT24 (p = .028), where the higher EIT resulted in higher dissemination. Comparisons at day 13 did not result in any statistical significance among treatments (Fig 2). Numerical infection and dissemination rates are given in S1 Table.

### Mosquito pool positivity and detection probability

All 8 pools that included a single positive mosquito body were positive for each duplicate (100%), indicating that a single mosquito in a pool of 100 mosquitoes in a surveillance setting is sufficient to detect ZIKV “activity” in this context.

### Simulation of locally acquired ZIKV infections in mosquitoes under temperature conditions

Table 1 shows the values of λ determined through non-linear least squares modeling using nls function in R and the maximum values for infection and disseminated (out of total infected) proportions from the experimental data. Fits of the experimental data according to estimates of lambda and 95% confidence limits are shown in S2 and S3 Figs, which demonstrate how well the parameter estimates of lambda (and thus the exponential rate in the model) follow the data. In the model, the transmission rate was set at 0, as the purpose was to identify establishment after a single generation of human-to-mosquito transmission (and not mosquito-to-human back to mosquito). In all simulations, there were no additional human infections other than the initial value of five, indicating the model is appropriate to assess mosquito-only infection kinetics.

**Table 1:** Parameter values by non-linear least squares (λ) and 95% confidence limits. Maximum proportion (p_max_) of infection or dissemination determined by the experimental data and binomial 95% confidence limits. (Disseminated infections are calculated out of total infected mosquitoes.).

Treatment	λ (95% CI)		p_MAX_ (95% CI)	
RT24-EIT24	λ_inf_	0.070 (0.044, 0.105)	p_inf.max_	0.57 (0.36, 0.77)
	λ_diss_	0.030 (0.007, 0.060)	p_diss.max_	0.46 (0.19, 0.73))
RT24-EIT28	λ_inf_	0.087 (0.069, 0.109)	p_inf.max_	0.73 (0.51, .96)
	λ_diss_	0.108 (0.063, 0.193)	p_diss.max_	0.73 (0.46, 0.99)
RT28-EIT24	λ_inf_	0.159 (0.133, 0.193)	p_inf.max_	0.89 (0.76, 1.0)
	λ_diss_	0.026 (0.023, 0.030)	p_diss.max_	0.29 (0.08, 0.51)
RT28-EIT28	λ_inf_	0.118 (0.112, 0.125)	p_inf.max_	0.79 (0.57, 1.0)
	λ_diss_	0.072 (0.042, 0.114)	p_diss.max_	0.73 (0.46, 0.99)

For each set of temperature conditions, there was between a 94.4% and 96.2% probability of at least one mosquito becoming exposed under model conditions and given an initial introduction of five infected humans into a naïve population (S4 Fig). Thus, the number of simulations that failed to produce any exposed mosquitoes was less than 6% for all treatment conditions. When examining the binomial confidence intervals of these probabilities, there were no differences among predicted probabilities of at least one exposed mosquito, indicating the model is appropriate to evaluate post-exposure kinetics (S4 Fig).

**Fig 3:**
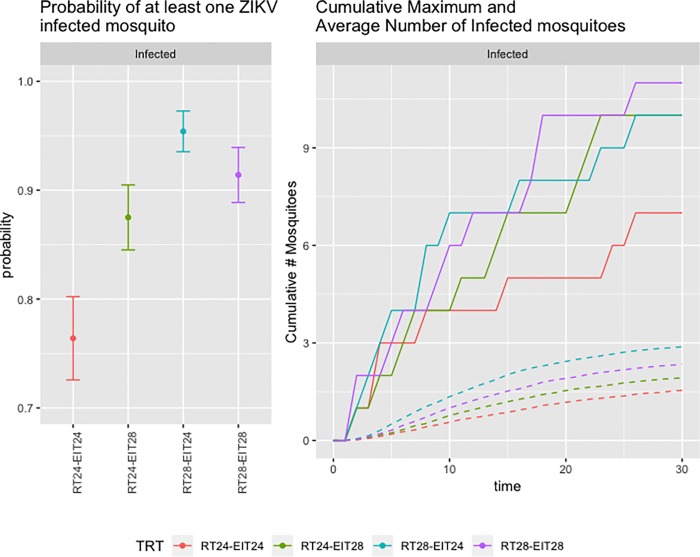
Left–The probabilities of at least one infected mosquito given at least one exposed mosquito following introduction of ZIKV infected humans into a naïve mosquito population. Bottom–The simulated cumulative maximum (solid lines) and average cumulative (dotted lines) number of infected mosquitoes over the course of 30 days following the introduction of 5 infectious humans into a naïve mosquito population.

To assess ZIKV kinetics within the naïve mosquito population, probabilities were calculated as the number of simulations where at least one mosquito became infected/disseminated divided by the total number of simulations where at least one mosquito had become exposed (as described above). There was between a 77.3% and 93.1% chance that ZIKV would successfully infect at least one mosquito (Fig 3). The temperature condition resulting in the lowest probability of infection was not surprisingly the RT24-EIT24 group (77.3%), while the highest chance of successful establishment was in the RT28-EIT24 group (93.1%). The overlap of 95% confidence intervals of RT28-EIT24 (93.1%) and RT28-EIT28 (90.2%) likely means this difference is due to stochastic processes. The RT24-EIT28 group had a probability of established infection of 84.2%. Overall, there was overlap of the 95% binomial confidence intervals among most treatments, signifying likely no significant pattern of infection success and temperature conditions. These results are summarized in S3 Fig.

When dissemination was predicted given at least one exposed mosquito, the highest probabilities were in the RT28-EIT28 group (46.7%) and the RT24-EIT28 group (43.8%). There was a moderate probability of dissemination in the RT28-EIT24 group (14.6%) and a low probability in the RT24-EIT24 group (4.1%). These results indicate that higher EITs are more important predictors of successful dissemination (S5 Fig, S3 Table).

The maximum number of mosquitoes at each time point was summed cumulatively over the 30 days modeled for each set of temperature conditions. Simulations predicted that the cumulative maximum number of infected mosquitoes ranged from 7 (RT24-EIT24) to 11 (RT28-EIT28) (Fig 3). The number of mosquitoes predicted to develop a disseminated infection ranged from 2 (RT28-EIT24) to 7 (RT24-EIT28) (S5 Fig). The average cumulative number of infected and disseminated mosquitoes is given in Fig 3 and S5 Fig, respectively, and was calculated as the average out of the total simulations resulting in at least one exposed mosquito.

When the maximum positivity of infection and dissemination (p_inf.max_ and p_diss.max_) were not included as a weight to the overall distribution of the infected and disseminated classes, there was a general increase in the probability of ZIKV infection establishment and thus likelihood of detection through surveillance, as well as the probability that locally exposed mosquitoes would eventually develop a disseminated infection (S3 Table). The differences in predicted infection establishment were modest, at most 9.5% under RT24-EIT24 conditions. The differences in dissemination were more marked (S3 Table). The largest difference was observed under RT28-EIT24 conditions when the difference in predicted probability of a disseminated infection developing was increased by 26.5% with the exclusion of p_inf.max_ and p_diss.max_.

## Discussion

After a mosquito takes a bloodmeal, ZIKV must establish infection in the midgut of the mosquito. The virus must then get past the midgut barrier in order to disseminate to the peripheral tissues. These are both critical first steps in the process of perpetuating transmission in naïve populations, as failure to infect and subsequently get past the midgut barrier means that mosquito will not be able to transmit. Temperature is a known driver of viral kinetics (infection and dissemination rates) within the mosquito and for many arboviruses, including ZIKV. In general, data suggest higher EITs lead to faster and higher rates of dissemination [13, 29, 30]. Less characterized is the role of larval temperature conditions on subsequent vector competence. While there is some lack of agreement among the effects of RT and EIT on the reported experimental ZIKV kinetics and the simulated infection probabilities across the four treatments, the prediction of dissemination success follows the tenet that higher EITs leads to more disseminated infections.

This lack of a consistent relationship between vector competence and RT and EIT has been observed in other arbovirus systems. In one study of DENV-1, it was shown that both EIT and RT affected infection and dissemination rates at 14 dpe, but in a non-uniform manner [28]. However, in another study, rearing temperature (24°C vs 28°C) did not significantly affect DENV-1 infection rates of *Ae*. *aegypti* at 17 dpe [27]. While not statistically significant, the results of this ZIKV investigation would seem to confirm the positive association of higher temperatures–especially during the EIP–with a trend towards higher and faster infection and dissemination rates [13, 29, 44]. But, importantly, it was demonstrated that ZIKV can infect and disseminate through mosquitoes at 24°C, and that temperature between juvenile and adults stages does not appear to alter vector competence.

Understanding the temporal nature of these processes compliments reporting of the magnitude of differences and can offer additional insights into the transmission dynamics of these viruses and is a useful and complimentary metric in describing within-host viral kinetics that is directly translatable to predictive models for sub-tropical and temperate regions of the world [40, 45–47]. This study demonstrated that inclusion of both the temporal component as well as the parameter describing maximum magnitude of these processes (infection and dissemination) is necessary for precise predictions and that failure to account for these qualities of the vector competence properties results in likely overestimation of infection establishment and dissemination. Future studies are needed to assess the interactions of larval and adult habitat conditions on the propagation of ZIKV through multiple transmission generations, including transmission rates and associated life-traits that may or may not be temperature and/or infection dependent [2]. In addition, there may be considerable effects of diurnal fluctuations in larval and adult development and thus competency for some viruses [29, 48–50]. However, the results herein offer insights into the relative importance of RT and EIT in the process of vector competence of ZIKV.

In conclusion, these results indicate that 1) there is no straightforward relationship between RT, EIT, and infection/dissemination rates, 2) in more temperate climates, ZIKV may still have the ability to establish in populations of *Aedes aegypti*, 3) despite an overall lack of significant differences in infection/dissemination rates, temperature may still alter the kinetics of ZIKV within the mosquito enough to affect the likelihood of infection establishment and detection within the context of mosquito surveillance programs, and 4) both the temporal and magnitude qualities of vector competence are necessary for parameterization of within-mosquito virus kinetics.

## Supporting information

S1 File Model parameters. These parameters are defined from aggregated data of dengue, a close and more characterized relative of ZIKV (compiled in [41]).
(DOCX)

S1 Table Infection and Dissemination Rates for each temperature condition. Dissemination is calculated (number positive legs)/(number infected).
(DOCX)

S2 Table Definition of transition rates between compartments of the stochastic SEIR model. (DOCX)

S3 Table Predicted probabilities of at least one infected mosquito and one disseminated infection given at least one locally exposed mosquito with and without the inclusion of p_max_ parameters. (DOCX)

S1 Fig Experimental Setup: Mosquito were reared at one constant temperature (either 24°C or 28°C). “Rearing Period” includes egg hatching, larval, pupal, and very young adult stages (up to 5 days post emergence). “Extrinsic Incubation Period (EIP)” refers to the time following ZIKV-infected blood-meal until sampling; the temperature associated with EIP (either 24°C or 28°C) is called the extrinsic incubation temperature (EIT).
(TIFF)

S2 Fig Mosquito infection rates (dots) and fitted distributions based on estimated rate parameter lambda (solid line) and upper and lower 95% confidence limits of lambda (dashed lines). (TIFF)

S3 Fig Mosquito dissemination rates (dots) and fitted distributions based on estimated rate parameter lambda (solid line) and upper and lower 95% confidence limits of lambda (dashed lines). (TIFF)

S4 Fig Probabilities of at least one exposed mosquito given the introduction of ZIKV infected humans into a naïve population. (TIFF)

S5 Fig **Left**–The probabilities of at least one disseminated mosquito given at least one exposed mosquito following introduction of ZIKV infected humans into a naïve mosquito population. **Right**–The simulated cumulative maximum (solid lines) and average cumulative (dotted lines) number of disseminated mosquitoes over the course of 30 days following the introduction of 5 infectious humans into a naïve mosquito population.
(TIFF)
