# Supplementary material for: Investigating the probability of establishment of Zika virus and detection through mosquito surveillance under different temperature conditions
Source: PLoS One. 2019 Mar 28;14(3):e0214306. doi: 10.1371/journal.pone.0214306 (PMC6438564; doi:10.1371/journal.pone.0214306)
Supplement: S1 File — These parameters are defined from aggregated data of dengue, a close and more characterized relative of ZIKV (compiled in [41]). (DOCX) [file pone.0214306.s001.docx]

**Supplemental Information File:**

**SI: Model parameters:** These parameters are defined from aggregated data of dengue, a close and more characterized relative of ZIKV (compiled in [41]).

a (biting rate) = 1 bite per day

z^-1^ (intrinsic incubation period) = 4 days

v^-1^ (recovery rate) = 9 days

ε (mosquito emergence rate) = 357.14 per day

μ^-1^ (average mosquito lifespan) = 18 days

q (average virus acquisition probability) = 48%

λ_inf_ (rate of mosquito becoming infected) = from data

λ_diss_ (rate of mosquito developing disseminated infection) = from data

λ_T_ (rate of mosquito becoming infectious) = 0

p_inf.max_ (maximum proportion infected) = from data

p_diss.max_ (maximum proportion disseminated) = from data
