# Supplementary material for: Investigating the probability of establishment of Zika virus and detection through mosquito surveillance under different temperature conditions
Source: PLoS One. 2019 Mar 28;14(3):e0214306. doi: 10.1371/journal.pone.0214306 (PMC6438564; doi:10.1371/journal.pone.0214306)
Supplement: S1 Table — Dissemination is calculated (number positive legs)/(number infected). (DOCX) [file pone.0214306.s002.docx]

S1 Table:

**S1 Table**: Infection and Dissemination Rates for each temperature condition. Dissemination is calculated (number positive legs)/(number infected).

| Treatment | DPI (n) | Infection (%) | Dissemination (%) |
| --- | --- | --- | --- |
| RT28 EIT28 | 7 (20) | 55 | 36.4 |
|  | 10 (17) | 70.6 | 41.7 |
|  | 13 (14) | 78.6 | 72.7 |
| RT28 EIT24 | 7 (35) | 62.9 | 18.2 |
|  | 10 (23) | 82.6 | 21.1 |
|  | 13 (19) | 89.5 | 29.4 |
| RT24 EIT24 | 7 (24) | 50 | 8.3 |
|  | 10 (25) | 44 | 18.2 |
|  | 13 (23) | 56.5 | 46.2 |
| RT24 EIT28 | 7 (20) | 45 | 66.7 |
