# Supplementary material for: Investigating the probability of establishment of Zika virus and detection through mosquito surveillance under different temperature conditions
Source: PLoS One. 2019 Mar 28;14(3):e0214306. doi: 10.1371/journal.pone.0214306 (PMC6438564; doi:10.1371/journal.pone.0214306)
Supplement: S2 Table — (DOCX) [file pone.0214306.s003.docx]

S2 Table

**S2 Table**: Definition of transition rates between compartments of the stochastic SEIR model.

| **Event** | **Change in state** | **Transition rate** |
| --- | --- | --- |
| Transmission from human to mosquito | (Sm, Em)🡪(Sm-1, Em+1) | aS_m_(qI_m_/N_h_) |
| Mosquito becomes infected | (Em, Im) 🡪(Em-1, Im+1) | (p_inf.max_λ_inf_)E_m_ |
| Mosquito develops disseminated infection | (Im, Dm) 🡪 (Im-1, Dm+1) | (p_diss.max_λ_diss_)I_m_ |
| Mosquito able to transmit | (Dm, Tm) 🡪 (Dm-1, Tm+1) | λ_T_D_m_ |
| Transmission from mosquito to human | (Sh, Eh)🡪 (Sh-1, Eh+1) | aS_h_(T_m_/N_h_) |
| Human becomes infectious | (Eh, Ih) 🡪 (Eh-1, Ih+1) | zE_h_ |
| Human recovery | (Ih, Rh) 🡪(Ih-1, Rh+1) | vI_h_ |
| Mosquito emergence | (Sm) 🡪 (Sm+1) | ε _m_ |
| Susceptible Mosquito death | (Sm)🡪(Sm-1) | μS_m_ |
| Exposed Mosquito death | (Em) 🡪 (Em-1) | μE_m_ |
| Infected Mosquito death | (Im) 🡪 (Im-1) | μI_m_ |
| Disseminated Mosquito death | (Dm)🡪 (Dm-1) | μD_m_ |
| Transmitting Mosquito death | (Tm)🡪(Tm-1) | μT_m_ |
