# Supplementary material for: Investigating the probability of establishment of Zika virus and detection through mosquito surveillance under different temperature conditions
Source: PLoS One. 2019 Mar 28;14(3):e0214306. doi: 10.1371/journal.pone.0214306 (PMC6438564; doi:10.1371/journal.pone.0214306)
Supplement: S3 Table — (DOCX) [file pone.0214306.s004.docx]

S3 Table

**S3 Table**: Predicted probabilities of at least one infected mosquito and one disseminated infection given at least one locally exposed mosquito with and without the inclusion of p_max_ parameters.

| Condition | p_max_ | P(Infection) | P(Dissemination) |
| --- | --- | --- | --- |
| RT24-EIT24 | Included | .764 | .191 |
|  | Excluded | .859 | .393 |
| RT24-EIT28 | Included | .875 | .630 |
|  | Excluded | .913 | .757 |
| RT28-EIT24 | Included | .954 | .235 |
|  | Excluded | .950 | .500 |
| RT28-EIT28 | Included | .914 | .585 |
|  | Excluded | .935 | .729 |
