# Supplementary figures and images for: Investigating the probability of establishment of Zika virus and detection through mosquito surveillance under different temperature conditions
Source: PLoS One. 2019 Mar 28;14(3):e0214306. doi: 10.1371/journal.pone.0214306 (PMC6438564; doi:10.1371/journal.pone.0214306)

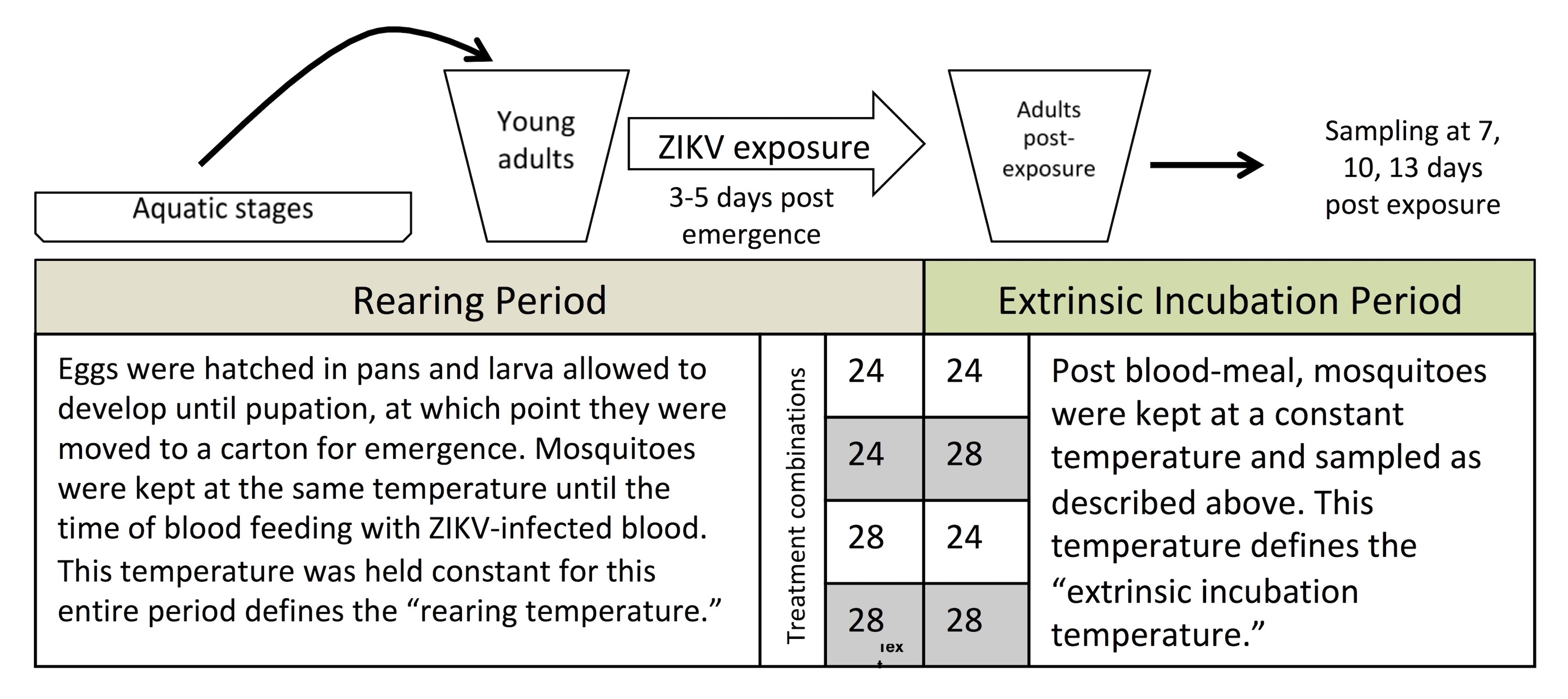

Supplement: S1 Fig — “Rearing Period” includes egg hatching, larval, pupal, and very young adult stages (up to 5 days post emergence). “Extrinsic Incubation Period (EIP)” refers to the time following ZIKV-infected blood-meal until sampling; the temperature associated with EIP (either 24°C or 28°C) is called the extrinsic incubation temperature (EIT). (TIFF) [file pone.0214306.s005.tiff]

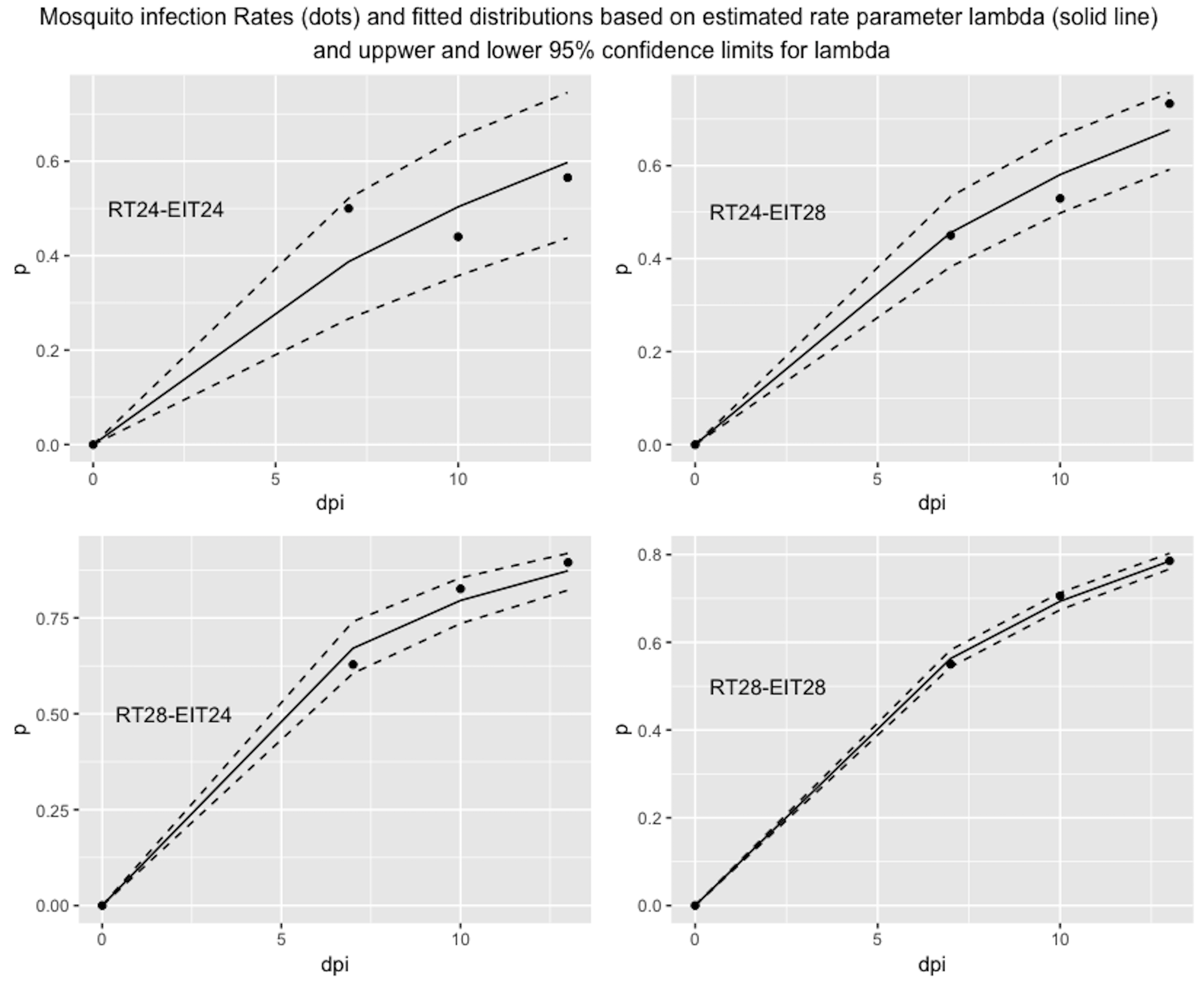

Supplement: S2 Fig — (TIFF) [file pone.0214306.s006.tiff]

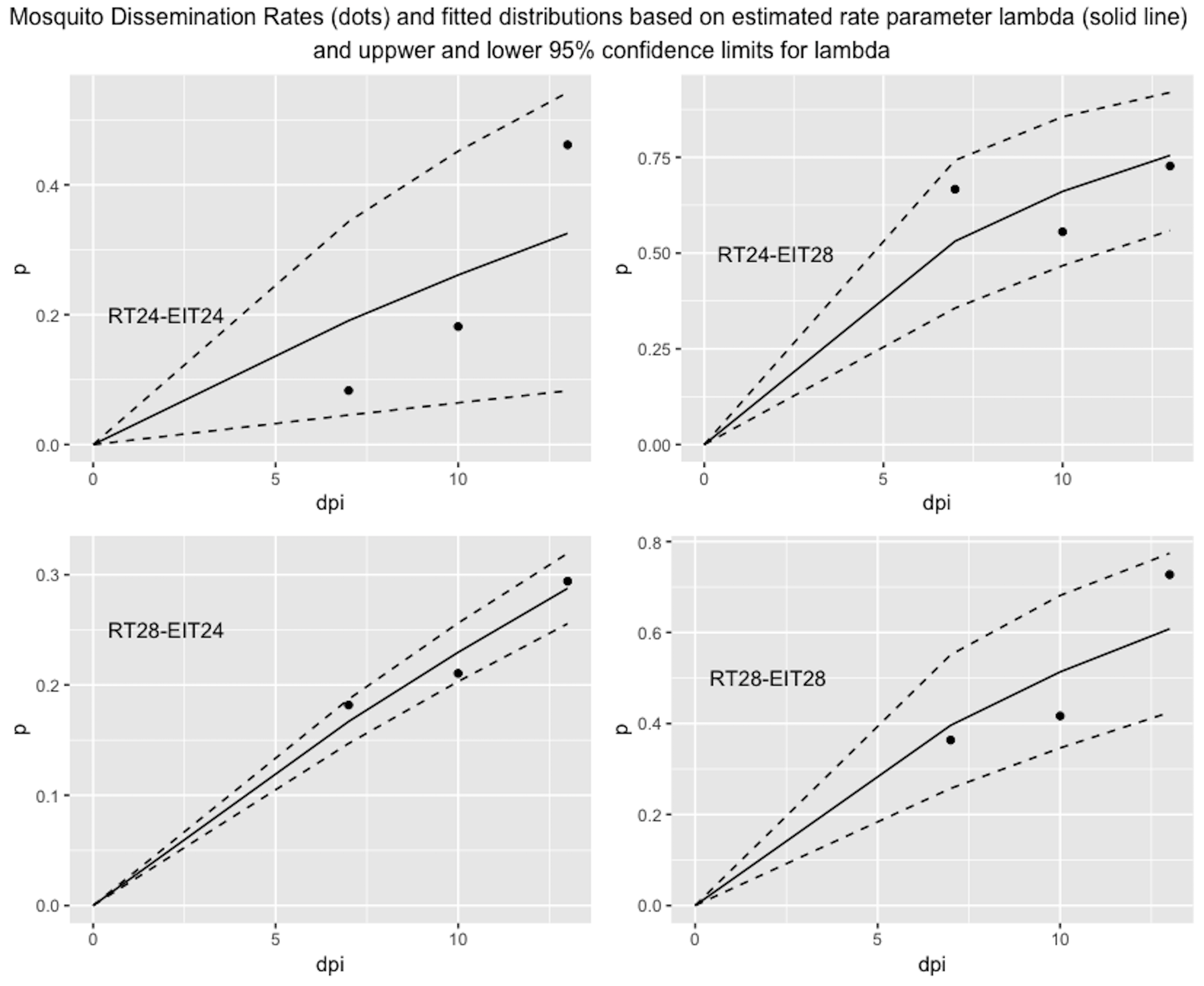

Supplement: S3 Fig — (TIFF) [file pone.0214306.s007.tiff]

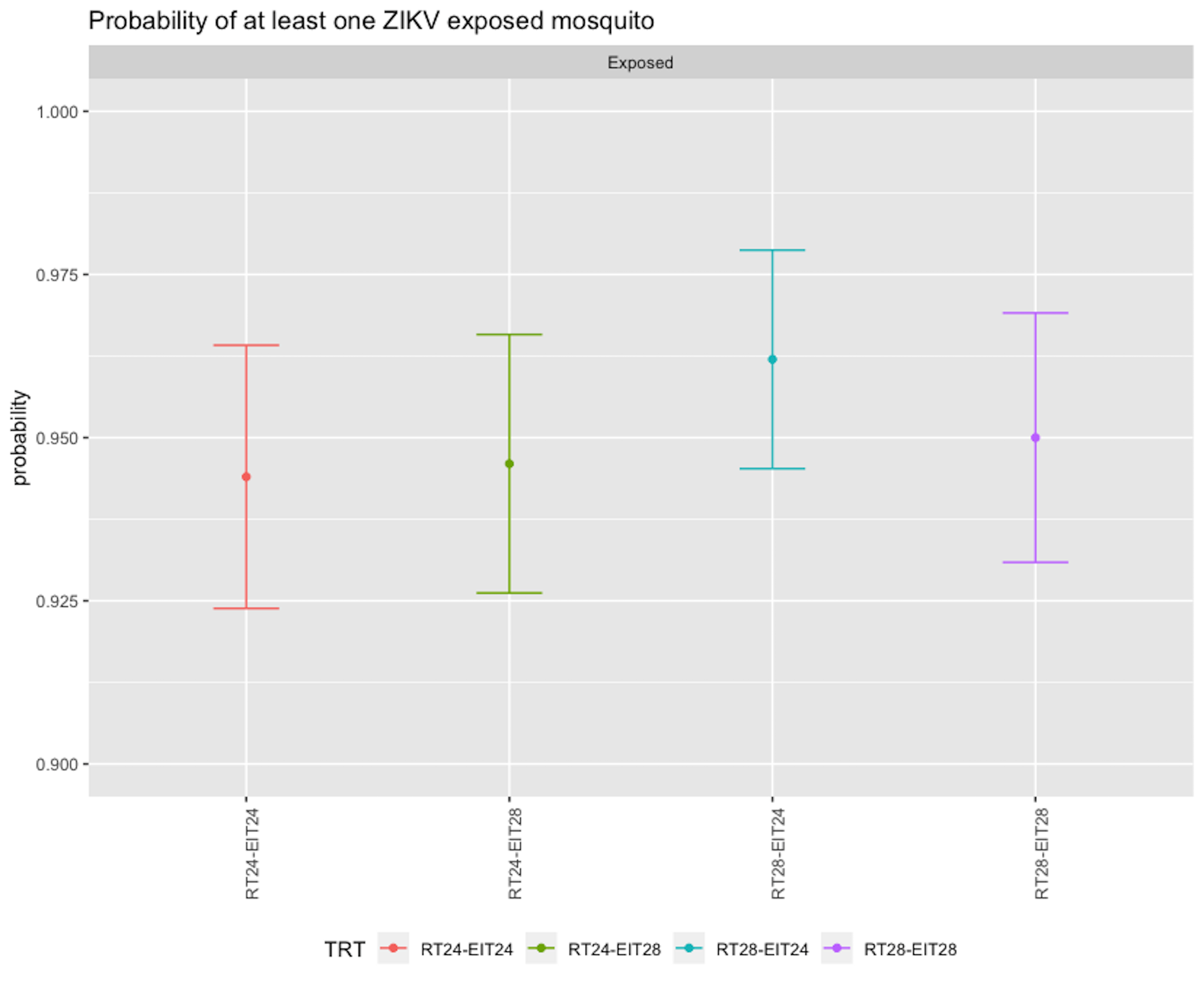

Supplement: S4 Fig — (TIFF) [file pone.0214306.s008.tiff]

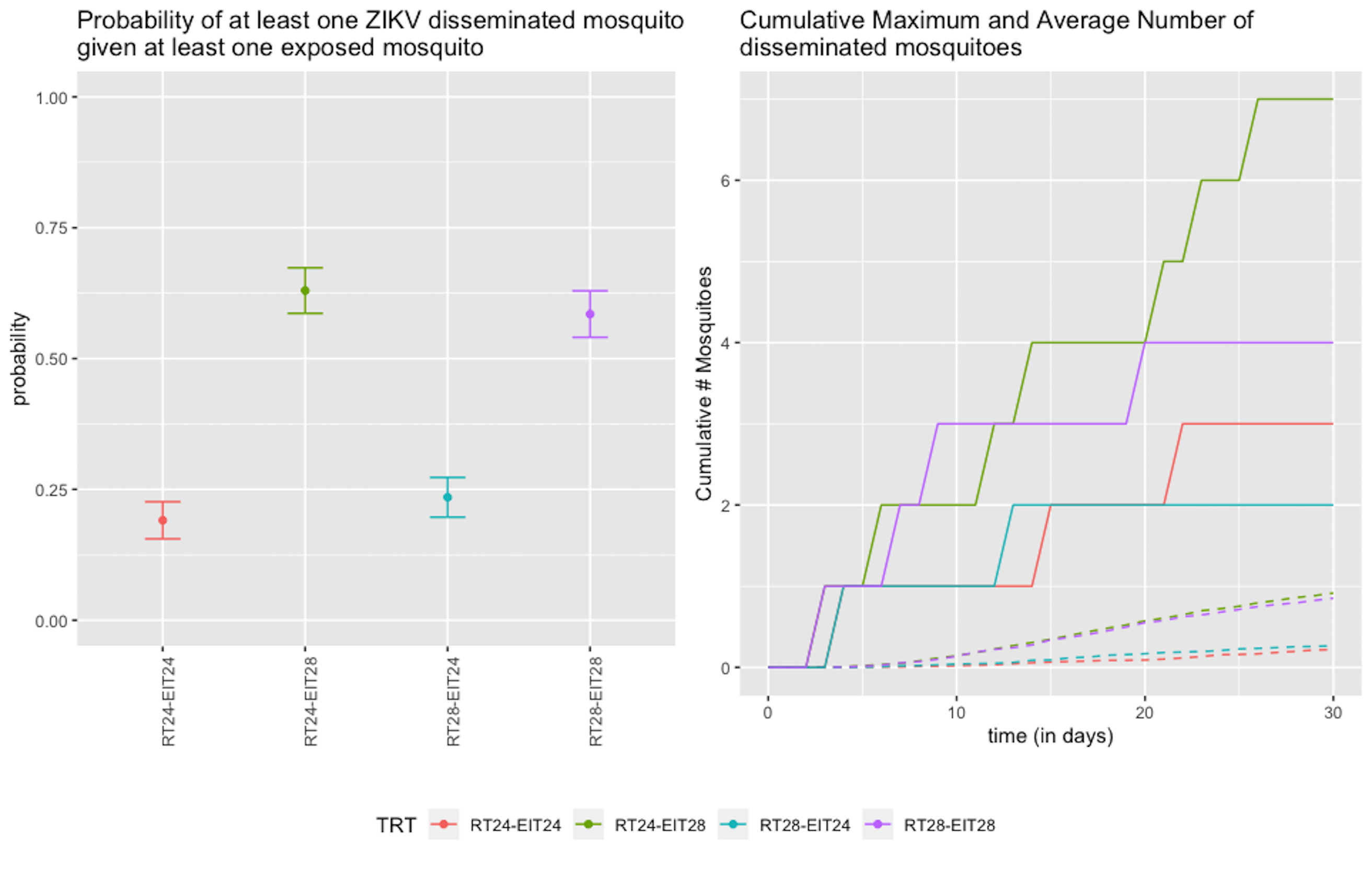

Supplement: S5 Fig — Left–The probabilities of at least one disseminated mosquito given at least one exposed mosquito following introduction of ZIKV infected humans into a naïve mosquito population. Right–The simulated cumulative maximum (solid lines) and average cumulative (dotted lines) number of disseminated mosquitoes over the course of 30 days following the introduction of 5 infectious humans into a naïve mosquito population. (TIFF) [file pone.0214306.s009.tiff]
